# From Clinical Practice to Basic Science, in Search for the Ideal Implantable Bioreactor: A Review on MFAT (Micro-Fragmented Adipose Tissue—Lipogems^®^) Biology and Its Clinical Applications

**DOI:** 10.3390/ijms27135710

**Published:** 2026-06-24

**Authors:** Carlo Tremolada, Giulio Alessandri

**Affiliations:** 1 Image Regenerative Clinic, 20122 Milan, Italy; giulio.alessandri@istitutoimage.com; 2 Image Regenerative Institute, 7500 Saint Moritz, Switzerland

**Keywords:** mesenchimal stem cells, clinical application, adipose tissue, micro-fragmention, tissue regeneration

## Abstract

The therapeutic use of adipose tissue has evolved from volumetric replacement to biologically driven regenerative applications. This brief review analyzes micro-fragmented adipose tissue (MFAT), obtained through Lipogems^®^ technology, as a paradigmatic example of reverse translation in regenerative medicine, where consistent clinical efficacy preceded and guided mechanistic investigation. Unlike enzymatic digestion or aggressive mechanical emulsification (nanofat), Lipogems^®^ device processing preserves the native stromal vascular niche, including extracellular matrix architecture and the pericyte–endothelial complex. We review the biological mechanisms underlying MFAT activity, compare MFAT with stromal vascular fraction (SVF) and nanofat, summarize consolidated clinical evidence across multiple medical and surgical specialties, and discuss emerging translational applications in oncology and sepsis. Collectively, the available data position MFAT as a structurally intact, biologically responsive tissue bioreactor bridging clinical practice and regenerative biology.

## 1. Introduction: The Reverse Translational Paradigm

Classical translational medicine traditionally follows a bench-to-bedside trajectory, where mechanistic discoveries precede clinical application. Micro-fragmented adipose tissue (MFAT) development via Lipogems technology represents a clear inversion of this model, in which reproducible clinical observations drove subsequent mechanistic investigation [1,2,3]. Early applications of adipose tissue grafting in plastic and reconstructive surgery demonstrated regenerative effects exceeding simple volumetric restoration, including scar softening, improved tissue elasticity, and recovery of radiation-damaged tissues [1]. These findings suggest that adipose tissue functions not merely as a filler but as a biologically active tissue, prompting systematic investigation into its intrinsic regenerative properties and stromal vascular niche [4,5,6,7,8,9,10,11,12,13,14,15].

## 2. The Technology: Preserving the Stromal Vascular Niche

Adipose tissue is the largest organ in the human body and represents a rich source of mesenchymal stromal cells (MSCs), pericytes, endothelial cells, and immune cells embedded within a complex extracellular matrix [1,6,7,8]. Adipose tissue has recently emerged as a privileged source of mesenchymal stem cells (MSCs). Due to their multipotent nature, these cells can differentiate in vitro into adipogenic, chondrogenic, osteogenic, and myogenic lineages. Their ability to secrete bioactive molecules defines them as true molecular “mini-pharmacies” [1,2,3,4,5], justifying their widespread use in orthopedic and reconstructive surgery. However, enzymatic isolation methods from lipoaspirate for obtaining stromal vascular fraction (SVF) and ex vivo expansion present critical issues: the risk of cellular senescence and loss of multipotency can compromise clinical efficacy, in addition to facing stringent regulatory constraints.

**Figure 1:**
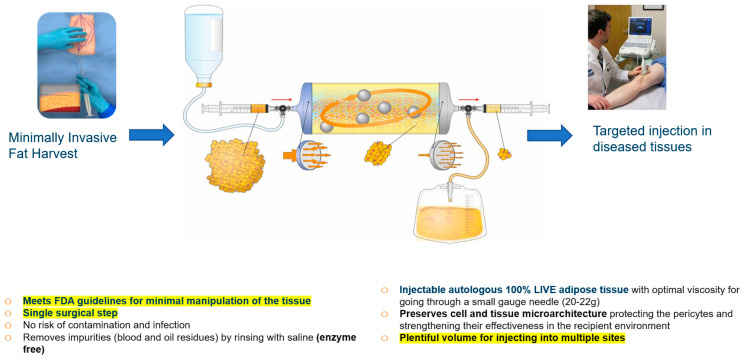
Adipose tissue is manually harvested with the blunt cannulas provided under local anesthesia with syringe suction. The 3 mm clusters of lipoaspirate are transferred in the lipogems device which allows for a gentle fragmentation to 0.3 mm, keeping the structure of the tissue almost completely intact and thoroughly washed from blood and oil impurities. The MFAT (lipogems) is collected in syringes and ready to be injected (usually under ultrasound guidance) in the recipient tissue working as a living graft for a long time. Proceedings of “Milan International Longevity Meeting 2025” presented by C. Tremolada (https://www.youtube.com/watch?v=8ASyr9xgp7o&t=5s Last accessed on 10 June 2026).

Therefore, innovative techniques were developed to obtain micro-fragmented adipose tissue (MFAT) with an intact stromal vascular niche and MSCs with high regenerative capacity. Among the different techniques, the Lipogems^®^ device, patented in 2010 and clinically available since 2013, demonstrated significant capacity to preserve the stromal vascular niche. In addition, Lipogems^®^ represents an intuitive system for the collection, processing, and grafting of micro-fragmented adipose tissue. This technology stands out for its high manageability and significant regenerative potential, supported by the presence of adipose tissue-derived MSCs [13,14,15]. Through a progressive washing and emulsion procedure, the size of the adipose aggregates is reduced to 0.3–0.8 mm. The final product (MFAT) preserves the pericytes within their original stromal vascular niche; once transplanted, these interact with the recipient site, activating as MSCs and triggering tissue repair processes. The Lipogems^®^ device for producing MFAT has been used in more than 70,000 patients worldwide in esthetic and reconstructive surgery, as well as in orthopedic and general surgery, with interesting and promising results and seemingly no drawbacks. Several clinical trials are on track to support the initial encouraging outcomes. The Lipogems^®^ device has emerged as a valid intraoperative system to obtain an optimal MFAT final product, preserving tissue microarchitecture, that may be used immediately for regenerative purposes [8,9] (Figure 1). Interestingly, to our knowledge, there appears to be no clinical research indicating the presence of harmful side effects in patients treated with MFAT.

### 2.1. Mechanical Processing Versus Enzymatic Digestion (Figure 2)

Enzymatic digestion using collagenase disrupts the extracellular matrix architecture and cell–cell interactions to isolate stromal vascular fraction (SVF), yielding a suspension of isolated cells devoid of native tissue organization [7,10]. In contrast, MFAT processing reduces adipose clusters to micro-units (of approximately 0.3–0.8 mm) while preserving adipocyte integrity, capillaries, connective tissue matrix, and perivascular structures [5,7,8,9,10]. This approach maintains the native microenvironment that governs cellular behavior and regenerative signaling and ensures a physiological and completely natural response [12,13,14,15].

Although comparative studies between MFAT and isolated MSCs are limited, current evidence highlights the biological superiority of MFAT. A specific study demonstrates that MFAT preserves a high density of NG2+ and CD146+ cells (pericytes) and microvascular endothelial cells. From a functional point of view, both MFAT and MSCs derived from these cells exert potent angiogenic and anti-inflammatory action. Their secretome inhibits the expression of adhesion molecules (ICAM-1 and VCAM-1), reduces U937 monocyte migration and adhesion, and suppresses pro-inflammatory chemokine release (RANTES and MCP-1). These data confirm the ability of MFAT to promote vascular stabilization and modulate the macrophage response [16]. In another study [17], analyzing MFAT clusters revealed that they are enriched in perivascular cells (i.e., mesenchymal stem cell–MSC ancestors) and in vitro analysis showed an increased release of growth factors and cytokines involved in tissue repair and regeneration compared to enzymatically derived MSCs. This suggests that the superior therapeutic potential of micro-fragmented adipose tissue is explained by a higher frequency of presumptive MSCs and enhanced secretory activity. Whether these added pericytes directly contribute to higher growth factors and cytokine production is not known. This clinically approved procedure allows for presumptive MSC transplantation without the need for expansion and/or enzymatic treatment, thus bypassing GMP guideline requirements and reducing the costs for cell-based therapies. In addition, cultured MSCs, obtained from the enzymatic digestion of LP, showed lower capacity to survive and to secrete regenerative molecules compared to MFAT when both were cultured under conditions that mimicked an unfavorable environment such as the environment found in vivo [18]. Although other studies are required to better clarify MFAT activity, the MSC contents retained in MFAT obtained through a mechanical process appear superior to MSCs isolated and cultured after enzymatic digestion.

**Figure 2:**
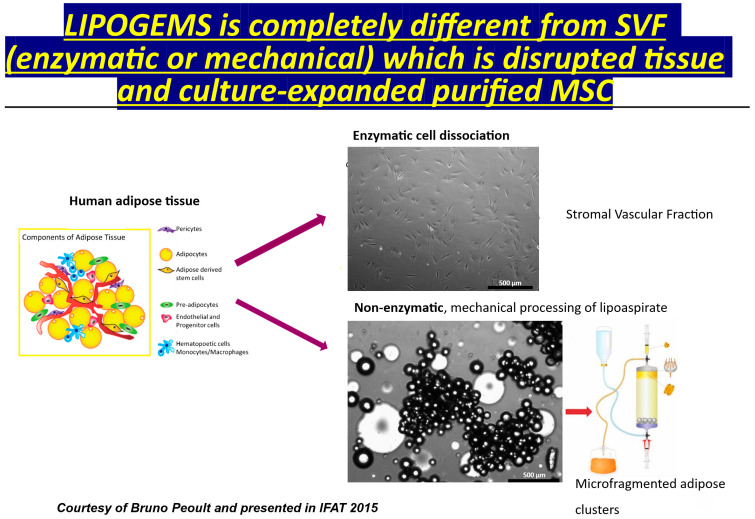
SVF is obtained by enzymatic or mechanical disruption of adipose tissue which loses its integrity while Lipogems maintains the microstructure of the tissue. Both can be used to produce nice stem cell cultures. Proceedings of “Milan International Longevity Meeting 2025” presented by C. Tremolada (https://www.youtube.com/watch?v=8ASyr9xgp7o&t=5s Last accessed on 10 June 2026).

### 2.2. MFAT as a Natural Implantable Bioreactor

**Figure 3:**
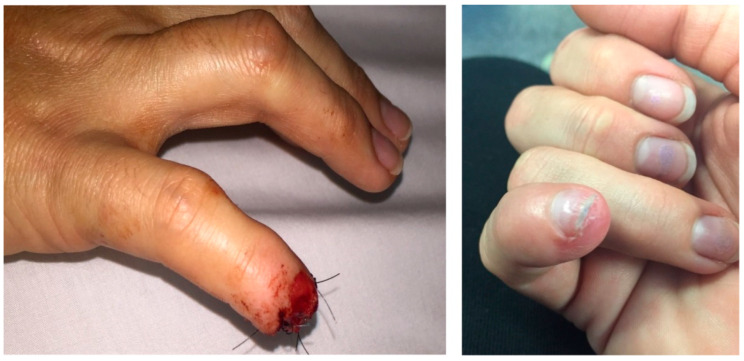
Finger regeneration in a 26-year-old man after injection of 0.5 mL of MFAT/Lipogems and clinical result at 8 months (this type of fingertip regeneration is the norm in young babies but not in adults). Proceedings of “Milan International Longevity Meeting 2025” presented by C. Tremolada, courtesy of Todd Malan, MD (https://www.youtube.com/watch?v=8ASyr9xgp7o&t=5s Last accessed on 10 June 2026).

Preserving tissue architecture allows MFAT to function as a natural autologous implantable bioreactor, integrating mechanical support, viable cells, and paracrine mediators within a living scaffold [10,13,14]. Micro-fragmentation increases the graft’s surface area, enhancing stromal vascular niche exposure and facilitating interaction with host tissues, ensuring a long-term effect [9,14]. This biological organization differentiates MFAT from isolated cell suspensions and underlies its reliability in in vivo clinical activity [6,10,11,13]. The microvascular density of the recipient tissue is clearly augmented by Lipogems tissue graft, as shown in histological samples of the vaginal corium of a patient previously treated with Lipogems [14]; this fact might explain why we consistently see clinical results that are typical of pediatric age such as meniscal [15] or fingertip regeneration (Figure 3) even in adults (Video S1; Supplementary Materials).

### 2.3. MFAT Versus Nanofat and Enzymatic-Derived SVF (Figure 4)

Nanofat is obtained through aggressive mechanical emulsification that intentionally disrupts adipocytes and the extracellular matrix, producing a liquid suspension suitable for superficial intradermal injections but that lacks structural scaffolding and cannot engraft [7,19,20]. These features make it impossible for nanofat to work as an implantable bioreactor in the long term. For this reason, it is difficult to compare its activity to that of MFAT in clinical applications, especially when both are used to regenerate complex tissue like cartilage [20]. Therefore, nanofat appears to be more similar to mechanical SVF than to MFAT [15,19,20,21,22,23]. Comparative analyses demonstrate that MFAT retains significantly higher pericyte content and secretory activity than enzymatically derived SVF [10,13,15,23]. MFAT exposes a significantly large amount of broken and intact capillaries and, consequently, of pericytes ready to function in a completely natural way [15]. Furthermore, it has been observed that MFAT actively produces microcapillaries in vitro in a similar way to the aorta ring (Figure 5) and also induces better vascularization in the recipient tissue [14], increasing tissue vascular density [12,23] and resulting in real tissue rejuvenation. This is evident clinically and by examining the histology of the tissues treated in gynecological and esthetic applications even years after surgical adipose grafting [12,13,14,15,23]. Longevity applications appear to be a clear possibility.

**Figure 4:**
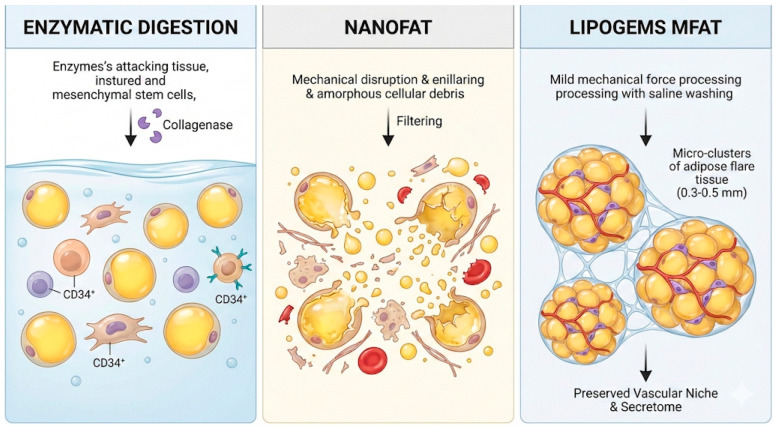
Enzymatic digestion of fat tissue by collagenase separates all cell types. Nanofat (which is a kind of mechanical SVF) mechanically destroys the adypocytes and spares the MSC but has leaves a lot of inflammatory cell debris behind. Lipogems MFAT is made of completely intact adipose clusters of about 0.3 mm and is free from cell debris. The microvessels are exposed on the surface and easily produce MSC after culture or in vivo when transplanted in the recipient tissue. Proceedings of “Milan International Longevity Meeting 2025” presented by C. Tremolada (https://www.youtube.com/watch?v=8ASyr9xgp7o&t=5s Last accessed on 10 June 2026).

**Figure 5:**
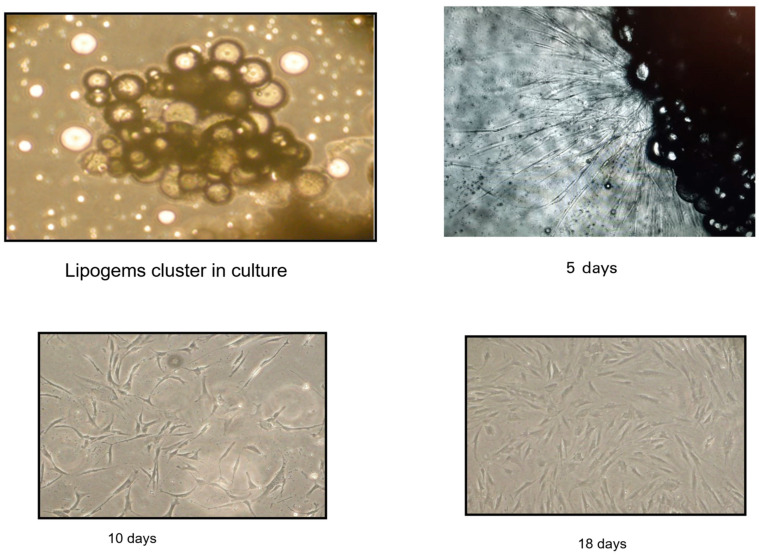
MAFT in culture. Fragments of MFAT in culture appear as clusters of luminescent adipocytes surrounded by several lipid droplets (**up left, 20× magnification**). After a few days of incubation, some fat fragments outgrew capillary-like structures (**up right, 10× magnification**). MSCs present in MFAT started to adhere and grow after 6–14 days of culture (**Low left, 20× magnification**) and reached confluence upon 18–25 days of culture (**low right, 20× magnification**). Proceedings of “Milan International Longevity Meeting 2025” presented by C. Tremolada (https://www.youtube.com/watch?v=&t=5s Last accessed on 10 June 2026).

## 3. Biological and Molecular Mechanisms: The Perivascular Niche

**Figure 6:**
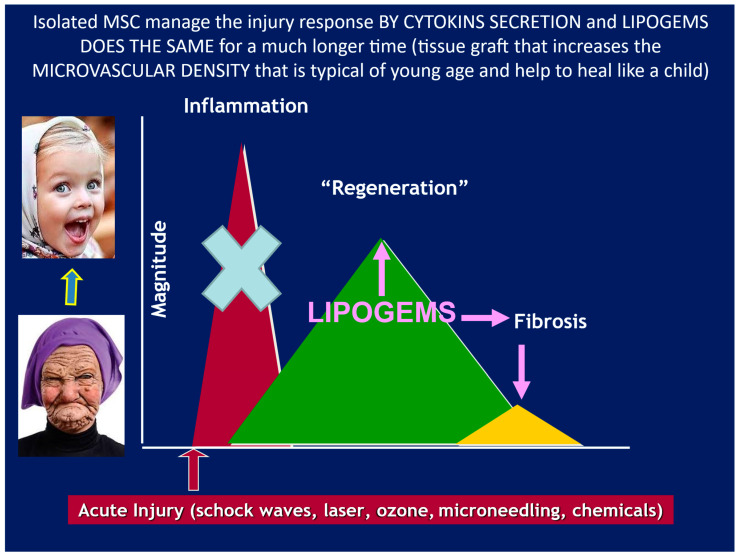
Regeneration of tissue is often started by mild controlled trauma (acute injury), which is a fact well known by wound surgeons. Lipogems is created by such mechanical trauma but most inflammatory cytokines are washed away in the waste bag (X on the schematic “red mountain”), which explains why there is strong early anti-inflammatory action clinically (only anti-inflammatory cytokines produced by activated MSC are present). When regeneration (schematic “green mountain”) is not completed, healing can proceed by the formation of scar tissue (schematic “yellow mountain”), and there is a delicate balance between these two healing mechanisms. Lipogems increases the content of cytokines involved in regeneration and decreases the content of cytokines involved in scarring, which is a typical response in patients of a young age and of well-vascularized healthy tissues. Proceedings of “Milan International Longevity Meeting 2025” presented by C. Tremolada (https://www.youtube.com/watch?v=8ASyr9xgp7o&t=5s Last accessed on 10 June 2026).

MFAT micro-clusters are enriched in intact and fragmented micro vessels surrounded by pericytes, which are now recognized as the in vivo precursors of MSCs [1,2,3,23]. Upon injury, inflammation, or mechanical stimulation, pericytes detach from the vascular wall and acquire an MSC-like phenotype, contributing to tissue repair through paracrine and immunomodulatory mechanisms [3,9,23]. The therapeutic effects of MFAT are therefore largely mediated by its secretome, including cytokines, growth factors, extracellular vesicles, and exosomes that modulate inflammation, angiogenesis, macrophage polarization, and tissue remodeling [3,4,6,8,9,10,11,12,13,14,15,22,23,24,25] (Figure 6). Focusing on MFAT in vivo activity, its molecular mechanism is quite complex because it likely involves a combination of MFAT intrinsic molecular activities (which are triggered by mechanical micro-fragmentation) and the inflammatory tissue microenvironment, where MFAT is usually applied in clinics. Looking at the MFAT secretome, data seems to indicate a significantly high content of angiogenic factors (such as Ang-1 and Ang-2; VEGF and MMP2 if compared to Ad-MSCs isolated through enzymatic digestion) [16]. In addition, MFAT produced significantly higher levels of G-CSF, SCGF-*β*, and HGF compared with non-micro-fragmented fat [17]. G-CSF production in MFAT could be associated with endothelium activation due to the shearing force produced by the Lipogems device. G-CSF has previously been shown to be a critical factor in augmenting the tissue regeneration of cartilage repair [26], in dermal and epidermal wound healing [27], and in rotator cuff healing and repair [28]. Moreover, G-CSF stimulates MSC production and activation, induces increased stem cell growth factor expression, HGF [29], and improved tissue recruitment capacity and anti-inflammatory status (IL-10 and TNF-*α* inhibition) [30]. Finally, the MFAT secretome did not contain inflammatory cytokines, but it has a potent anti-inflammatory property by reducing migration, monocyte adhesion to the endothelium, and RANTES and MCP-1 inflammatory chemokine secretion [16,17]. Taken together, the results seem to indicate that the MFAT secretome is able to stimulate tissue regeneration and concomitantly block several important monocyte inflammatory functions. In conclusion, MFAT may be effective because it combines a natural structural scaffold organization with a very well-preserved SVF and extended MSC survival in a hostile inflammatory microenvironment [17,18]. However, further studies are essential to understand how MFAT acts in vivo. This may be a complex investigation, but it is necessary to ameliorate its clinical use, which is one of the main purposes of this review.

## 4. Clinical Applications

### 4.1. Orthopedics and Musculoskeletal Disorders

MFAT has been widely applied in degenerative musculoskeletal conditions, particularly osteoarthritis of the knee, hip, and shoulder, but also in spine facets and foot and hand joints, showing potential for cartilage regeneration [23,24,25]. Observational studies reported significant pain reduction and functional improvement following intra-articular MFAT injection [31,32,33,34,35,36,37,38,39,40,41,42,43,44,45,46,47,48,49,50]. This clinical improvement appears after 3 months, and improvement continues for well over a year. Comparative studies demonstrated outcomes comparable or superior to bone marrow aspirate concentrate (BMAC), with sustained benefits at mid- and long-term follow-up [31]. Randomized controlled trials and large real-world series have further confirmed the safety and efficacy of MFAT compared with saline or platelet-based orthobiologics and also show clinical benefits in advanced AO cases [44,51,52,53]. Preclinical and translational data suggest that MFAT promotes biologically regulated cartilage remodeling rather than direct chondrogenic replacement. Repeated use may be necessary in more advanced cases, and Lipogems tissue cryopreservation may help in the development of a simpler clinical protocol, avoiding repeated adipose tissue harvesting. The use of cryopreserved Lipogems will also facilitate scientific validation, potentially allowing for an easier double blinding of clinical trial protocols and simplifying potential ethical issues (it avoids sham fat harvesting and potentially allows for secondary crossover) [54,55].

### 4.2. Wound Healing and Proctology

In general surgery and proctology, MFAT has shown efficacy in treating ischemic and diabetic wounds [56,57,58,59,60], complex anal fistulas [61,62], rectovaginal fistulas, and fecal incontinence, showing promising anatomical and functional muscular regeneration [63]. Clinical studies report favorable safety profiles and meaningful functional improvement, attributed to the combined volumetric support and biological stimulation of angiogenesis and re-epithelialization [64]. The long-term benefit of this innovative approach has been demonstrated [65,66], and endoscopic and minimally invasive approaches further extend their applicability in complex refractory cases [66,67]. Using MFAT as a natural scaffold and drug delivery system may help in the development of innovative topical medical devices for wound healing [68].

### 4.3. Esthetic and Reconstructive Medicine

In esthetic and reconstructive applications, MFAT improves skin texture, elasticity, and tissue quality. Clinical experience from thousands of patients over more than a decade consistently describes an early anti-inflammatory effect with minimal swelling, even in combined surgery followed by progressive tissue remodeling and long-term optimization of the skin and subcutaneous tissue [1,8,15,23,69].

## 5. Emerging Translational Frontiers

### 5.1. Oncology: MFAT as a Drug Delivery System

Beyond regenerative medicine, MFAT has emerged as a potential local drug delivery system for lipophilic chemotherapeutic agents [70,71,72,73,74,75,76]. In this regard, our group first discovered that the fresh preparation of MFAT specimens and, surprisingly, even its devitalized MFAT (DMFAT) counterpart (a fat derivative obtainable through a simple freezing/thawing (F/T) procedure), were very effective in adsorbing and releasing significant amounts of the anti-cancer molecule Paclitaxel (PTX). Both MFAT and DMFAT loaded with PTX (MFAT-PTX; DMFAT-PTX) were able to kill many different human cancer cell lines in vitro when they located nearby tumor cells, with impressive long-lasting anti-cancer activity. In addition, DMFAT-PTX placed in nude mice at the site of surgical resection of neuroblastoma (NB) orthotopic xenografts blocked or delayed tumor relapse, thus confirming its potent anti-tumor activity and suggesting its possible use as a DD tool in treating human cancer [70]. Subsequently, the antitumor efficacy of MFAT and DMFAT loaded with PTX has been validated in hepatocellular carcinoma (HCC) models. In vitro tests (2D and 3D) on Hep-3B cells confirmed marked antiproliferative activity. In vivo, a single subcutaneous administration of DMFAT-PTX near the tumor induced significant growth inhibition, with 33% complete regressions. Histological and pharmacokinetic analyses demonstrated sustained release of PTX at the tumor site, with a high rate of apoptosis. These findings suggest DMFAT-PTX as a promising tool for the local chemotherapy of advanced HCC or inoperable tumors [73].

Therapeutic potential has been further confirmed in the veterinary field: a case of multilocal mesothelioma in a dog was managed with 17 intracavitary injections of MFAT-PTX over 22 months. The treatment was found to be safe and well tolerated, ensuring a long-lasting clinical improvement. The pharmacokinetic profile showed low systemic absorption and persistence in the extravascular compartments, validating the loco-regional approach for different oncological pathologies [72]. Finally, a recent study involved a single intra-tumoral treatment of gliomas in dogs using DMFAT combined with PTX. The study showed no short- or mid-term adverse effects. The quality of life score was good in most cases. MRI scans and a 3D rendering assessment revealed a decrease in tumor volume in five out of six dogs treated with DMFAT-PTX, whereas all three dogs in the control group that were treated with temozolomide or lomustine showed continuous tumor volume increase. Histological analysis demonstrated that DMFAT-PTX caused necrosis of the tumoral mass and a reactive glial–mesenchymal response, with no signs of neurotoxicity in the brain tissue apart from the treated focal tumor site. In summary, these preliminary results suggest that DMFAT-PTX treatment may be a promising approach for glioma treatment in dogs, warranting further investigation with a larger group of canine patients. Moreover, these findings provide a proof of concept, demonstrating the safety and feasibility of this approach for potential human translational applications [71,74].

In conclusion, these preclinical and clinical data suggest that MFAT can act as a biodegradable scaffold capable of sustained local drug release for a long period (for up to two months in vitro) while reducing systemic toxicity. Translational models in mesothelioma, hepatocellular carcinoma, and brain tumors suggest that MFAT-based delivery may overcome the limitations of conventional chemotherapy, including poor tissue penetration and systemic adverse effects. In addition, MFAT retains the capacity to deliver anti-cancer drugs even after devitalization (obtained upon a freezing and thawing procedure), leading to the possibility for multiple treatments [70,71,72,73,74,75,76].

### 5.2. Infection and Sepsis (and Exosomes)

Experimental evidence indicates that MSCs exhibit organ-protective properties and improve the survival rate in sepsis models [77,78,79,80,81,82,83,84,85,86,87,88,89,90,91,92,93,94,95,96]. Interestingly, MSCs have been shown to activate macrophages which possess potent immunomodulatory ability and therapeutic potential in sepsis [82,83]. This macrophage activation was shown to occur via the cyclo-oxygenase (COX)-2/prostaglandin pathway in septic patients [81]. MSCs may therefore help control the inflammatory response at the acute phase of severe sepsis and improve outcomes. MSCs are routinely obtained enzymatically from fat lipoaspirate (LA) as stromal vascular fraction (SVF) and, in most cases, undergo further ex vivo expansion. In addition to the high cost, the large number of processing steps make its use difficult in an emergency context. Lipogems^®^ (LG) is an innovative and commercially available system that provides micro-fragmented fat in vivo in a short time (about 20 min), without expansion or enzymatic treatment. Using this technology, fat can be micro-fragmented and washed from pro-inflammatory oil and blood residues while preserving viable elements with perivascular identity within an intact stromal vascular niche. Based on this premise, a study performed by A. Bougle et al. [92] showed that in a murine model of severe sepsis, the intraperitoneal injection of micro-fragmented fat improved early inflammatory status and outcomes, at least in part, by a cyclo-oxygenase-2-mediated mechanism [92]. However, the potential therapeutic value of MFAT in severe sepsis warrants further investigation.

Indeed, to date, most studies have employed mesenchymal cells or their derivatives produced in culture (secretome). Currently, extracellular vesicles (EVs) or exosomes obtained from MSC cultures appear to be of great interest for potential clinical applications in infectious diseases [93,94,95,96,97]. However, the potential therapeutic use of EVs/exosomes directly produced by MFAT requires further investigation. It is possible, however, that in the near future, an MFAT source for regenerative factor production will be developed and studied in both preclinical and clinical settings. In particular, MFAT-derived EVs can be obtained rapidly (EVs are widely present in the washing bags obtained during the micro-fragmentation process to obtain Lipogems tissue) and therefore may be isolated/prepared and used directly in an operating room. In addition, MFAT-derived EVs have low production costs because, unlike those obtained from MSCs, they do not require culture or cell expansion.

## 6. Conclusions

Micro-fragmented adipose tissue represents a distinct paradigm in regenerative medicine, in which preserving native tissue architecture enables biological activity that cannot be replicated by isolated cells or enzymatically derived products [1,11,23]. Lipogems^®^ technology exemplifies a reverse translational pathway in which consistent clinical efficacy guided mechanistic discovery, leading to a deeper understanding of the stromal vascular niche and its therapeutic potential. Beyond established applications in orthopedics, surgery, and esthetic medicine, emerging evidence in oncology and inflammatory diseases highlights MFAT as a dynamic, living therapeutic platform with broad translational relevance, used both as a direct bioreactor and as a local drug delivery system or as a source of exosomes [97] for potential cell-free regenerative applications. 

## Data Availability

No new data were created or analyzed in this study. Data sharing is not applicable to this article.

## Supplementary Materials

The following supporting information can be downloaded at https://www.mdpi.com/article/10.3390/ijms27135710/s1.
